# Sex, sexual orientation, and gender identity data collection across electronic health record platforms: a national cross-sectional survey

**DOI:** 10.1093/jamiaopen/ooae127

**Published:** 2024-11-04

**Authors:** Carl G Streed, Maylene Navarra, Lauren B Beach, Gregory Phillips, Paige N Hackenberger, Sumanas Jordan

**Affiliations:** Section of General Internal Medicine, Boston University Chobanian and Avedisian School of Medicine, Boston, MA 02118, United States; GenderCare Center, Boston Medical Center, Boston, MA 02118, United States; GenderCare Center, Boston Medical Center, Boston, MA 02118, United States; Institute for Sexual and Gender Minority Health and Wellbeing, Northwestern University, Chicago, IL 60611, United States; Department of Medical Social Sciences, Northwestern University Feinberg School of Medicine, Chicago, IL 60611, United States; Department of Preventive Medicine, Northwestern University Feinberg School of Medicine, Chicago, IL 60611, United States; Institute for Sexual and Gender Minority Health and Wellbeing, Northwestern University, Chicago, IL 60611, United States; Department of Medical Social Sciences, Northwestern University Feinberg School of Medicine, Chicago, IL 60611, United States; Department of Preventive Medicine, Northwestern University Feinberg School of Medicine, Chicago, IL 60611, United States; Institute for Sexual and Gender Minority Health and Wellbeing, Northwestern University, Chicago, IL 60611, United States; Division of Plastic and Reconstructive Surgery, Northwestern University Feinberg School of Medicine, Chicago, IL 60611, United States; Institute for Sexual and Gender Minority Health and Wellbeing, Northwestern University, Chicago, IL 60611, United States; Division of Plastic and Reconstructive Surgery, Northwestern University Feinberg School of Medicine, Chicago, IL 60611, United States

**Keywords:** electronic health record, sexual orientation, gender identity

## Abstract

**Objectives:**

To assess the current state of sex, sexual orientation, and gender identity (SSOGI) data collection options in US electronic health record (EHR) platforms.

**Materials and Methods:**

We utilized an anonymous survey distributed via purposive snowball sampling to assess EHR platforms across the United States.

**Results:**

Of 90 surveys started, 41 (45.6%) were completed and used for data analysis. Respondents represented a geographically diverse sample of health care centers across the United States. EPIC was the most used EHR platform (70.7%) followed by Cerner (9.8%). Across reported platforms, a majority utilized structured fields to collect and document patient SSOGI data (*n* = 25, 61.0%). There was variability across platforms regarding SSOGI data elements collected. No platform collected all recommended SSOGI data elements.

**Discussion:**

Significant variation exists across EHR platforms and across health care settings using the same EHR platform.

**Conclusion:**

National standards need to be followed for SSOGI data collection in EHR platforms.

## Introduction

Researchers and clinicians rely on data from electronic health records (EHRs) to monitor the health of populations and address the specific needs of patients.^1^ Despite established health disparities^2^ and the designation of sexual and gender minority (SGM) populations of special interest to the NIH,^3^ there is inconsistent documentation of patient sex, sexual orientation, and gender identity (SSOGI) within EHRs.^4^^,^^5^ Efforts have been made to incorporate SSOGI data in EHRs by federal agencies, such as the Centers for Medicare & Medicaid Services and the Office of the National Coordinator of Health Information Technology.^6^^,^^7^ Further, in 2016, the Human Resources Services Administration Bureau of Primary Health Care began requiring federally funded community health centers to collect and provide SSOGI data as part of their annual Uniform Data Systems report.^8^ These entities as well as the Affordable Care Act have motivated the collection of SSOGI patient data in EHRs.^5^^,^^9–11^

Sex, sexual orientation, and gender identity data collection and documentation are a key component of enhancing meaningful dialogue during clinical encounters and promote the provision of high-quality care. Patient–provider discussions about SSOGI can facilitate a more accurate assessment of self-reported health and behaviors.^12^ Further, by routinely eliciting SSOGI data using a structured format, EHR systems are better equipped to notify health care providers of appropriate and targeted care and preventive services.^13^ Maintaining and utilizing SSOGI information in the EHR can promote communication among staff within health care organizations, improving delivery of care and patient satisfaction.^8^ Further, SSOGI data capture can be used in concert with other data collection tied to social determinants to support a more patient-centered and comprehensive approach to patient care. Additional downstream systems can also be accurately initiated to direct care through such mechanisms as best practice advisories, and laboratory systems could provide the appropriate reference range for patients and clinicians based on SSOGI data relevant to the clinical scenario. Yet, despite patients being willing to report SSOGI in the EHR,^5^^,^^9^^,^^14^ some health care organizations do not collect these data.^14^^,^^15^ Even among health care organizations willing to implement SSOGI data collection, there are limitations due to inconsistent guidance.^4^^,^^16^

A recent report from the the National Academies of Science, Engineering, and Medicine (NASEM) has provided baseline recommendations for SSOGI data collection in a variety of scenarios.^1^ However, there remains no standardized requirement for what components of SSOGI data should be included in EHR platforms. Without clear guidance, the various EHR platforms and their health care system clients are left to develop their own SSOGI data collection methods; there are potentially as many SSOGI data collection methods as there are EHR platforms.

For 2022, EPIC had over a third (35.9%) of the hospital market followed by Oracle Cerner (24.9%), which translates to nearly three quarters (73.4%) of all hospital beds.^17^ Therefore, exploring the largest EHR platforms, by market share and hospital bed coverage, we can have a better understanding of the landscape of SSOGI data collection in clinical settings in the United States. The following study provides a snapshot of existing EHR platforms and assesses the breadth and depth of their SSOGI data collection capabilities.

## Methods

### Survey design

The survey instrument was developed by the study team and pilot-tested among peer clinicians and researchers before distribution. The survey assessed if and how data were collected for the following domains: sexual orientation, gender identity, sex assigned at birth, legal sex, intersex status, organ inventory, and diagnostic codes to identify transgender and intersex patients; definitions of these concepts and terms are integrated into the survey. Response options were presented as “check all that apply” along with free text response. There was the option to provide a screenshot of an empty template for collecting SSOGI information from their EHR platform. We also asked if patients had agency to edit their SSOGI information via patient portal or similar interface (survey in Supplementary Material).

### Survey respondents

Participants were eligible if they were age 18 or older, spoke English, could use a computer with internet connection, worked in a clinical setting, and had access to their institution’s EHR system. Participants were initially recruited through study team members’ professional networks via email. After initial recruitment via professional networks, new survey respondents were recruited for participation through purposive snowball sampling with a goal of achieving EHR platform, geographic, and institutional diversity. We distributed survey invitations with 2 follow-up emails to nonresponders between May 2022 and December 2022. We recorded participant institution, participant role, and institution location. Survey respondents were monitored and no duplicate respondents were encountered during data collection. Purposive sampling was used to exclude additional respondents from the same institution as long as a completed survey had been submitted. Survey respondents were not otherwise excluded from study participation. Only completed surveys were considered in the final analysis. The Boston University Institutional Review Board reviewed the research and determined it was exempt (IRB #H-42532).

Descriptive statistics were used to characterize the data. Sex, sexual orientation, and gender identity data collection measures were evaluated based on recommendations from the NASEM 2022 report on measuring sex, gender identity, and sexual orientation.^1^ At the time of the study, there was no required standard on how SSOGI data should be collected, and we therefore utilized the NASEM recommendations as a baseline comparison (Supplementary Material).

## Results

**Figure 1.**
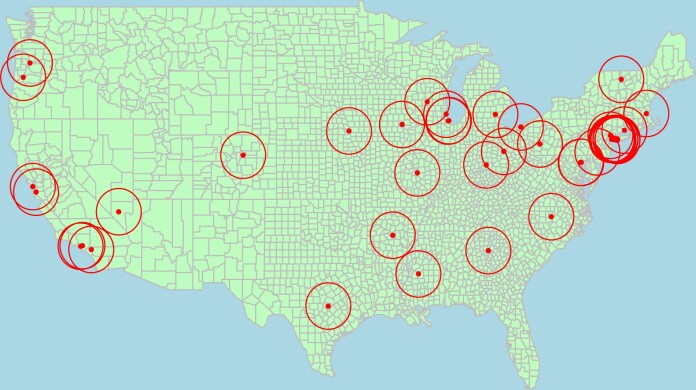
Health care centers described by survey respondents with 100 mile radii.

Of 90 surveys initiated, 41 (45.6%) were completed. Respondents represented a geographically diverse sample of health care centers across the United States, with the greatest concentration in the northeastern United States (Figure 1). A total of 8 distinct EHR platforms were represented through survey responses. EPIC was the most commonly used EHR platform among respondents (70.7%) followed by Cerner (9.8%).^17^

Across reported platforms, a majority noted utilizing structured fields to collect and document patient SSOGI data (*n* = 25, 61.0%). There was significant variability across platforms regarding specific SSOGI data elements collected (Table 1). No platform collected all SSOGI data elements per NASEM criteria.

**Table 1.** Characteristics of SSOGI data collection by EHR platforms surveyed.

Response categories	Total *N* = 41, *n* (%)	EPIC *n* = 29, *n* (%)	Cerner *n* = 4, *n* (%)	AthenaHealth *n* = 1, *n* (%)	AllScript *n* = 1, *n* (%)	Meditech *n* = 2, *n* (%)	GE Centricity *n* = 1, *n* (%)	VHA *n* = 1, *n* (%)	Another EHR *n* = 2, *n* (%)
SSOGI data collection uses structured data fields	20 (48.7)	17 (58.6)	0 (0)	1 (100.0)	0 (0)	0 (0)	1 (100.0)	1 (100.0)	0 (0)
Sexual orientation collected	21 (51.2)	18 (62.0)	0 (0)	1 (100.0)	0 (0)	0 (0)	1 (100.0)	1 (100.0)	0 (0)
Sexual orientation options									
Lesbian or gay	21 (51.2)	18 (62.0)	0 (0)	1 (100.0)	0 (0)	0 (0)	1 (100.0)	1 (100.0)	0 (0)
Straight (not gay or lesbian)	16 (39.0)	13 (44.8)	0 (0)	1 (100.0)	0 (0)	0 (0)	1 (100.0)	1 (100.0)	0 (0)
Bisexual	20 (48.8)	17 (58.6)	0 (0)	1 (100.0)	0 (0)	0 (0)	1 (100.0)	1 (100.0)	0 (0)
Two-spirit	0 (0)	0 (0)	0 (0)	0 (0)	0 (0)	0 (0)	0 (0)	0 (0)	0 (0)
I use a different term (free text)	10 (24.4)	8 (27.6)	0 (0)	1 (100.0)	0 (0)	0 (0)	0 (0)	1 (100.0)	0 (0)
Don’t know	8 (19.5)	7 (24.1)	0 (0)	1 (100.0)	0 (0)	0 (0)	0 (0)	0 (0)	0 (0)
Prefer not to answer	11 (26.8)	11 (37.9)	0 (0)	0 (0)	0 (0)	0 (0)	0 (0)	0 (0)	0 (0)
Sexual orientation options not in NASEM									
Asexual	5 (12.2)	5 (17.2)	0 (0)	0 (0)	0 (0)	0 (0)	0 (0)	0 (0)	0 (0)
Pansexual	6 (14.6)	6 (20.7)	0 (0)	0 (0)	0 (0)	0 (0)	0 (0)	0 (0)	0 (0)
Queer	7 (17.1)	7 (24.1)	0 (0)	0 (0)	0 (0)	0 (0)	0 (0)	0 (0)	0 (0)
Questioning	3 (7.3)	3 (10.3)	0 (0)	0 (0)	0 (0)	0 (0)	0 (0)	0 (0)	0 (0)
Same gender loving	0 (0)	0 (0)	0 (0)	0 (0)	0 (0)	0 (0)	0 (0)	0 (0)	0 (0)
Omnisexual	1 (2.4)	1 (3.4)	0 (0)	0 (0)	0 (0)	0 (0)	0 (0)	0 (0)	0 (0)
Intersex data is collected	3 (7.3)	2 (6.9)	0 (0)	0 (0)	0 (0)	0 (0)	0 (0)	1 (100.0)	0 (0)
Intersex status is asked separate from sex assigned at birth	1 (2.4)	1 (3.4)	0 (0)	0 (0)	0 (0)	0 (0)	0 (0)	0 (0)	0 (0)
Is gender identity data collected?	25 (61.0)	19 (65.5)	1 (25.0)	1 (100.0)	0 (0)	0 (0)	1 (100.0)	1 (100.0)	2 (100.0)
Gender identity options									
Female	22 (53.7)	17 (58.6)	1 (25.0)	1 (100.0)	0 (0)	0 (0)	1 (100.0)	1 (100.0)	1 (50.0)
Male	22 (53.7)	17 (58.6)	1 (25.0)	1 (100.0)	0 (0)	0 (0)	1 (100.0)	1 (100.0)	1 (50.0)
Transgender	23 (56.1)	19 (65.5)	1 (25.0)	1 (100.0)	0 (0)	0 (0)	1 (100.0)	1 (100.0)	0 (0)
Two-spirit	2 (4.9)	1 (3.4)	0 (0)	0 (0)	0 (0)	0 (0)	0 (0)	0 (0)	1 (50)
I use a different term (free text)	12 (29.3)	9 (31.0)	0 (0)	1 (100.0)	0 (0)	0 (0)	1 (100.0)	1 (100.0)	0 (0)
Don’t know	1 (2.4)	0 (0)	0 (0)	0 (0)	0 (0)	0 (0)	0 (0)	0 (0)	1 (50.0)
Prefer not to answer	12 (29.3)	12 (41.4)	0 (0)	0 (0)	0 (0)	0 (0)	0 (0)	0 (0)	0 (0)
Gender identity options not in NASEM									
Nonbinary	16 (39.0)	14 (48.2)	0 (0)	1 (100.0)	0 (0)	0 (0)	1 (100.0)	0 (0)	0 (0)
Genderfluid	1 (2.4)	1 (3.4)	0 (0)	0 (0)	0 (0)	0 (0)	0 (0)	0 (0)	0 (0)
Third gender	2 (4.9)	2 (6.9)	0 (0)	0 (0)	0 (0)	0 (0)	0 (0)	0 (0)	0 (0)
Queer	1 (2.4)	1 (3.4)	0 (0)	0 (0)	0 (0)	0 (0)	0 (0)	0 (0)	0 (0)
Questioning	2 (4.9)	2 (6.9)	0 (0)	0 (0)	0 (0)	0 (0)	0 (0)	0 (0)	0 (0)
Sex assigned at birth is asked separate from gender identity	22 (53.7)	17 (58.6)	1 (25.0)	1 (100.0)	1 (100.0)	0 (0)	1 (100.0)	1 (100.0)	0 (0)
Sex assigned at birth options									
Male	24 (58.5)	18 (62.0)	2 (50.0)	1 (100.0)	0 (0)	0 (0)	1 (100.0)	1 (100.0)	1 (50.0)
Female	24 (58.5)	18 (62.0)	2 (50.0)	1 (100.0)	0 (0)	0 (0)	1 (100.0)	1 (100.0)	1 (50.0)
Don’t know	14 (34.1)	13 (44.8)	0 (0)	1 (100.0)	0 (0)	0 (0)	0 (0)	0 (0)	0 (0)
Prefer not to answer	12 (29.3)	12 (41.4)	0 (0)	0 (0)	0 (0)	0 (0)	0 (0)	0 (0)	0 (0)
Organ inventory	17 (41.5)	16 (55.17)	0 (0)	0 (0)	0 (0)	0 (0)	1 (100.0)	0 (0)	0 (0)
Patients can update SSOGI via patient portal	16 (39.02)	15 (51.72)	0 (0)	0 (0)	0 (0)	0 (0)	1 (100.0)	0 (0)	0 (0)
Persons with access to patient SSOGI									
Clinicians (eg, MD, DO, NP, PA, etc.)	26 (63.4)	18 (62.1)	2 (50.0)	1 (100.0)	1 (100.0)	1 (50.0)	1 (100.0)	1 (100.0)	1 (50.0)
Nurses	23 (56.1)	17 (58.6)	2 (50.0)	1 (100.0)	1 (100.0)	1 (50.0)	1 (100.0)	0 (0)	0 (0)
Front desk staff	22 (53.7)	16 (55.2)	1 (25.0)	1 (100.0)	1 (100.0)	1 (50.0)	1 (100.0)	1 (100.0)	0 (0)
Registration	20 (48.8)	15 (51.7)	1 (25.0)	1 (100.0)	1 (100.0)	1 (50.0)	1 (100.0)	0 (0)	0 (0)
Social workers	23 (56.1)	17 (58.6)	1 (25.0)	1 (100.0)	1 (100.0)	1 (50.0)	1 (100.0)	1 (100.0)	0 (0)
Researchers	17 (41.5)	13 (44.8)	1 (25.0)	1 (100.0)	0 (0)	1 (50.0)	1 (100.0)	0 (0)	0 (0)
Medical students	21 (51.2)	16 (55.2)	1 (25.0)	1 (100.0)	1 (100.0)	1 (50.0)	1 (100.0)	0 (0)	0 (0)
Someone not listed	4 (9.8)	2 (6.9)	0 (0)	0 (0)	0 (0)	1 (50.0)	0 (0)	0 (0)	1 (50.0)
Decline to answer	0 (0)	0 (0)	0 (0)	0 (0)	0 (0)	0 (0)	0 (0)	0 (0)	0 (0)
Respondent trained on entering SSOGI in EHR									
Yes, through current institution	19 (46.3)	14 (48.3)	1 (25.0)	0 (0)	0 (0)	1 (50.0)	1 (100.0)	1 (100.0)	1 (50.0)
Yes, from an outside organization or a previous institution	4 (9.8)	3 (10.3)	0 (0)	0 (0)	0 (0)	1 (50.0)	0 (0)	0 (0)	0 (0)
No	11 (26.8)	6 (20.7)	3 (75.0)	1 (100.0)	1 (100.0)	0 (0)	0 (0)	0 (0)	0 (0)
I don’t know	1 (2.4)	0 (0)	0 (0)	0 (0)	0 (0)	0 (0)	0 (0)	0 (0)	1 (50.0)

Abbreviations: EHR, electronic health record; NASEM, National Academies of Science, Engineering, and Medicine; SSOGI, sex, sexual orientation, and gender identity; VHA, Veteran Health Administration.

Sex assigned at birth was recorded as a distinct field by 53.7% of EHRs surveyed (*n* = 22). Sexual orientation was collected by 51.2% of our sample (*n* = 21), with the most frequently offered selections of “lesbian or gay” (*n* = 21, 51.2%), “bisexual” (*n* = 20, 48.8%), and “straight (not gay or lesbian)” (*n* = 16, 39.0%). Gender identity was collected by 61.0% (*n* = 25). Most commonly offered selections were “transgender” (*n* = 23, 56.1%), “female” (*n* = 22, 53.7%), and “male” (*n* = 22, 53.7%). Anatomy inventory documentation fields were offered by 41.5% (*n* = 17), and patients were able to self-report SSOGI via patient portal or similar means in 14 EHRs (31.4%). Additional sexual orientation and gender identity categories and frequency of their presence in EHRs are noted in Table 1.

Infrequently collected SSOGI elements included data on two-spirit identity and details of differences of sex development (DSD)/intersex designations. Only 1 surveyed EHR collected two-spirit data (2.4%); this was nested within structured selections as part of gender identity. Three EHRs collected intersex data (7.3%), with only 1 (2.4%) collecting this distinctly from the sex assigned at birth field. No surveyed platforms had detailed questions regarding DSD/intersex status.

## Discussion

Of all centers surveyed, just over half (51.2%) had EHR platforms capable of documenting sexual orientation, nearly two-thirds (61%) were capable of documenting gender identity.

Over a decade has passed since the World Professional Association for Transgender Health (WPATH) first published recommendations highlighting the need for standardized collection of SSOGI information within EHR platforms.^18^ Subsequently, federal rules and regulations have required SSOGI data collection within EHR platforms.^6–8^ Despite these, our study suggests current methods to collect and record structured SSOGI data remain highly variable across EHR platforms and, in fact, largely absent. Specifically, our results from a geographically diverse sample of health care centers across the United States utilizing a variety of EHR platforms notes that no EHR platform collects all elements of SSOGI as recommended by NASEM.^1^

Collection of SSOGI data requires EHR capability as well as prioritization of SGM patient care. Asking patients to disclose sensitive information must be met with an institutional promise of meaningful action to protect their privacy, honor their identity, and promote their individual health. Clinicians and researchers alike must demand that SSOGI-related data collection implementations and interventions remain centered on bioethical principles (beneficence, nonmaleficence, autonomy, and justice) to minimize additional discrimination that SGM patients already face within healthcare.^4^^,^^18–20^

Implementation of SSOGI data collection necessitates universal elements. The capabilities and limitations of each EHR platform must be considered. The role of community stakeholders must be clearly outlined, and their guidance should be incorporated throughout the process. Prior to implementing SSOGI data collection, stakeholders must determine how data will apply to other elements of the health system—how it will affect clinical, administrative, and research workflows. Additionally, staff must be educated about proper collection methods to improve competence and promote patient trust. Further, as terminology, legislation, and technology pertaining to SGM populations are in constant flux,^21^ protocols for continual assessment and adjustment must keep up with evolving needs. As more states (23 states and DC as of April 2024) are allowing additional legal sex designations beyond “M” or “F”, the need for EHRs to collect and interpret nonbinary sex and gender data rises to the forefront.^22^

Our study evaluated SSOGI data collection compared to the NASEM criteria.^1^ However, even these thoughtful recommendations are incomplete in some areas; the exclusion of pronouns and preferred name collection from NASEM highlights room for improving patient-facing elements of data collection.

Our data suggest that many fields are often left out of standardized SSOGI collection. Those most frequently excluded were two-spirit status, intersex status, and discrete fields for organ/anatomic inventory. Additional categories for sexual orientation and gender identity not noted in the NASEM report but are frequently recommended in community standards were also absent, including asexual, queer, and nonbinary. Furthermore, language such as “other” to capture identities not available as a discrete field selection can be stigmatizing and can ostracize certain SGM identities. These notable gaps and faux-pas are clear opportunities for immediate improvement across all health systems.

While SGM status may be derived from several elements within the medical record (eg, diagnosis codes, keyword text mining), structured SSOGI data capture is viewed as the most accurate and ethical.^23^ Of all platforms surveyed, nearly two-thirds (63.4%) had structured data fields for sexual orientation and 61% for gender identity. Only a third (39.0%) afforded patients the option to self-report this information in their medical record. Through self-report options, SSOGI data collection from patients promotes autonomy and leads to higher data accuracy.

While our study confirms that many EHRs remain incapable of collecting SSOGI, recent research demonstrates reporting mandates may address this. In a 2016 report of all of 1367 US health care centers caring for 25 860 296 patients in the United States and territories, sexual orientation and gender identity data were missing for 77.1% and 62.8% of patients, respectively.^15^ By 2021, sexual orientation and gender identity data were missing for 29.1% and 24.0% of patients, respectively.^24^

Our study highlights the opportunities for improved SSOGI collection across all EHR platforms and corresponding health systems. With accurate, dynamic measurement, health systems can track and intervene on health disparities in real-time. For example, during the COVID-19 pandemic, institutions with established SSOGI data collection were able to assess the impact on SGM patient health and tailor messaging and treatment recommendations accordingly.^25^

Federal priorities^26^ and the current National Institute of Health strategic plan^27^ emphasize health equity and SGM population health. These initiatives provide benchmarks by which to measure quality outcomes in SGM populations. Adoption of SSOGI collection standards will allow health systems to remain at the forefront of these priority areas and respond to quality standards and new opportunities as they arise.

### Limitations

This study was limited by data collection through survey response. To capture as many different EHR systems as possible, surveys were distributed via exponential discriminative snowball sampling. Consequently, survey respondent selection was not randomized and may be skewed toward providers who work in systems with an emphasis on SGM health and/or have implemented SSOGI data collection. Additionally, survey response rate could not be calculated and differences between survey respondents and nonrespondents could not be studied. Despite limitations from survey distribution method, surveys asked about EHR system elements and not about respondent personal details, thoughts, or opinions. As such, referral patterns which skew toward certain personal characteristics of survey respondents are unlikely to confound findings regarding SSOGI data collection within EHR platforms.

## Conclusion

Health care systems remain unequipped to adequately collect SSOGI as recommended and required. No one EHR platform meets a minimum of standards as defined by NASEM guidance on SSOGI data collection. The tools to adequately collect SSOGI exist but remain underutilized. This remains a missed opportunity to assess and address SGM population health.

## Supplementary Material

ooae127_Supplementary_Data

## Data Availability

The data underlying this article cannot be shared publicly due to the privacy of the health care systems that participated in the study. The data will be shared on reasonable request to the corresponding author following ethical review and execution of appropriate data use agreements.
