# Supplementary material for: Sex, sexual orientation, and gender identity data collection across electronic health record platforms: a national cross-sectional survey
Source: JAMIA Open. 2024 Nov 4;7(4):ooae127. doi: 10.1093/jamiaopen/ooae127 (PMC11534177; doi:10.1093/jamiaopen/ooae127)
Supplement: Supplementary file 1 [file ooae127_supplementary_data.docx]

EHR SOGI Data Collection

Start of Block: Electronic Health Record Questionnaire

|  |
| --- |

We are asking you to share information as part of a research collaborative to assess and improve measures of sex, sexual orientation, and gender identity (SSOGI). This research is part of a National Institutes of Health funded study (1R01AA029076-01), Project Recognize: Improving Measurement of Alcohol Use and Other Disparities by Sex, Sexual Orientation, and Gender Identity through Community Engagement. **As part of this study, we are asking you to fill out this survey to see how sex, sexual orientation, and gender identity data are collected in the electronic medical record (EMR) system at your institution.**

There are no anticipated risks to you and no direct benefits for participating in this survey. Your participation will inform best practices in collecting SSOGI data. Based on piloting, we expect your participation will take approximately 7-10 minutes. There is no payment for completion. We will not record your name or any personal identifying information. There is no consequence for choosing to not participate in the study

The investigator or a member of the research team will try to answer all of your questions. If you have
questions or concerns at any time, contact May Navarra, maylene.navarra@bmc.org, Senior Research Assistant at Boston Medical Center. 

| Page Break |  |
| --- | --- |

What is your institution? (You can fill this form for another institution if you work at multiple institutions with EMR systems)  Please avoid using abbreviations.

________________________________________________________________

What EMR system does your institution use?

- Epic  (1) 

- AllScripts  (2) 

- Praxis EMR  (3) 

- eClinical Works  (4) 

- Cerner  (5) 

- NextGen  (6) 

- Athenahealth  (7) 

- GE Centricity  (8) 

- Meditech  (9) 

- Greenway Health  (10) 

- Veteran Health Affairs (VHS)  (11) 

- Another EMR system (Please specity)  (12) __________________________________________________

Please share a screenshot or other file format (e.g., PDF, docx, etc.) of an empty template for collecting sexual orientation, gender identity information from your EMR system.

End of Block: Electronic Health Record Questionnaire

Start of Block: Sexual Orientation

Sexual orientation refers to the person's self-identified pattern of attraction to other persons (e.g. asexual, bisexual, heterosexual, homosexual, etc)

Does this EMR system collect data about sexual orientation using structured data fields (e.g., clickable fields)?

- Yes  (1) 

- No  (2) 

- Unsure  (3) 

- Decline to answer  (4) 

Skip To: End of Block If Does this EMR system collect data about sexual orientation using structured data fields (e.g., cl... = No

Skip To: End of Block If Does this EMR system collect data about sexual orientation using structured data fields (e.g., cl... = Decline to answer

What response options are available when collecting sexual orientation? (check all that apply)

- Asexual  (1) 

- Bisexual  (2) 

- Gay  (3) 

- Lesbian  (4) 

- Pansexual  (5) 

- Queer  (6) 

- Questioning  (7) 

- Same-gender loving  (8) 

- Straight/heterosexual  (9) 

- Other (with free text)  (10) 

- Other (with no free text)  (11) 

- My EMR includes another sexual orientation category (please specify)  (12) __________________________________________________

End of Block: Sexual Orientation

Start of Block: Gender Identity

Gender identity refers to someone's self-identification along a spectrum of male or female or something else (e.g., genderqueer, man, woman, transgender man, transgender woman, etc).

Does this EMR system collect data about Gender Identity using structured data fields (e.g., clickable fields)?

- Yes  (1) 

- No  (2) 

- Unsure  (3) 

- Decline to Answer  (4) 

Skip To: End of Block If Does this EMR system collect data about Gender Identity using structured data fields (e.g., click... = No

Skip To: End of Block If Does this EMR system collect data about Gender Identity using structured data fields (e.g., click... = Decline to Answer

What response options are available when collecting gender identity? (check all that apply)

- Genderqueer/non-binary/gender non-conforming  (1) 

- Man  (2) 

- Transgender man  (3) 

- Transgender woman  (4) 

- Woman  (5) 

- Other (with free text)  (6) 

- Other (with no free text)  (7) 

- My EMR includes another gender identity category (please specify)  (8) __________________________________________________

End of Block: Gender Identity

Start of Block: Sex Assigned at Birth

Does this EMR system explicitly collect data about sex assigned at birth using structured data fields (e.g., clickable fields)?

- Yes  (1) 

- No  (2) 

- Unsure  (3) 

- Decline to answer  (4) 

Skip To: End of Block If Does this EMR system explicitly collect data about sex assigned at birth using structured data fi... = No

Skip To: End of Block If Does this EMR system explicitly collect data about sex assigned at birth using structured data fi... = Decline to answer

What response options are available when collecting sex assigned at birth? (check all the apply)

- Male  (1) 

- Female  (2) 

- Intersex  (3) 

- Something else (please specify)  (4) __________________________________________________

End of Block: Sex Assigned at Birth

Start of Block: Legal Sex

Legal sex refers to the sex marker on a person's government documents such as a birth certificate or a drivers license.

Does this EMR system collect data on legal sex using structured data fields (e.g., clickable fields)?

- Yes  (1) 

- No  (2) 

- Unsure  (3) 

- Decline to answer  (4) 

Skip To: End of Block If Does this EMR system collect data on legal sex using structured data fields (e.g., clickable fiel... = No

Skip To: End of Block If Does this EMR system collect data on legal sex using structured data fields (e.g., clickable fiel... = Decline to answer

What response options are available when collecting legal sex? (check all that apply)

- Male  (1) 

- Female  (2) 

- Intersex  (3) 

- Non-Binary  (4) 

- Something else  (5) __________________________________________________

End of Block: Legal Sex

Start of Block: Intersex

Intersex is a general term used for a variety of conditions in which a person is born with a reproductive or sexual anatomy that doesn’t seem to fit the typical definitions of female or male. For example, a person might be born appearing to be female on the outside, but having mostly male-typical anatomy on the inside.

Does this EMR system collect intersex data using structured data fields (e.g., clickable fields)?

- Yes  (1) 

- No  (2) 

- Unsure  (3) 

- Decline to answer  (4) 

Skip To: End of Block If Does this EMR system collect intersex data using structured data fields (e.g., clickable fields)? = No

Skip To: End of Block If Does this EMR system collect intersex data using structured data fields (e.g., clickable fields)? = Decline to answer

What response options are available when asking if a person is intersex? (check all that apply)

- Yes  (1) 

- No  (2) 

- Unsure  (3) 

- Something else  (4) __________________________________________________

End of Block: Intersex

Start of Block: Diagnostic Codes

Does your EMR system rely on diagnostic codes/categories to identify transgender patients?

- Diagnostic codes only  (1) 

- Structured fields only  (2) 

- Both diagnostic codes and structured fields  (3) 

- I don't know  (4) 

- Something else  (5) __________________________________________________

Does your EMR system rely on diagnostic codes/categories to identify intersex patients?

- Diagnostic codes only  (1) 

- Structured fields only  (2) 

- Both diagnostic codes and structured fields  (3) 

- I don't know  (4) 

- Something else  (5) __________________________________________________

End of Block: Diagnostic Codes

Start of Block: Organ Inventory

An organ inventory allows patients to select what body parts they have and how to refer to them.

Does this EMR system collect data on which organs the patient has using structured data fields (e.g., clickable fields)?

- Yes  (1) 

- No  (2) 

- Unsure  (3) 

- Decline to answer  (4) 

Skip To: End of Block If Does this EMR system collect data on which organs the patient has using structured data fields (e... = No

Skip To: End of Block If Does this EMR system collect data on which organs the patient has using structured data fields (e... = Decline to answer

What response options are available when collecting data on organs the patient has? (check all that apply)

- Breast or breast tissue  (1) 

- Cervix  (2) 

- Ovaries  (3) 

- Penis/phallus  (4) 

- Prostate  (5) 

- Testicles  (6) 

- Uterus/ womb  (7) 

- Vagina/ frontal genital opening  (8) 

- Another organ  (9) __________________________________________________

End of Block: Organ Inventory

Start of Block: Privacy

Who has access to seeing patient's sexual orientation and gender identity information? (check all that apply)

- Clinicians (e.g., MD, DO, NP, PA, etc.)  (1) 

- Nurses  (2) 

- Front desk staff  (3) 

- Registration  (4) 

- Social Workers  (5) 

- Reasearchers  (6) 

- Medical Students  (7) 

- Someone not listed  (8) __________________________________________________

- I don't know  (9) 

- Decline to answer  (10) 

What is your role at this institution? (check all that apply)

- Clinician (e.g., MD, DO, NP, PA, etc.)  (1) 

- Nurse  (2) 

- Front desk staff  (3) 

- Registration  (4) 

- Social Worker  (5) 

- Researcher  (6) 

- Medical Student  (7) 

- Something else  (8) __________________________________________________

- Decline to answer  (9) 

Have you received training on entering SSOGI (sex / sex characteristics, sexual orientation and gender identity) information on the EMR? (select all that apply)

- Yes, through my current institution  (1) 

- Yes, from an outside organization or a previous institution  (2) 

- No  (3) 

- I don't know  (4) 

What gender information is available upon first opening patients' charts? (e.g. summary sheet, sidebar, etc.) (check all that apply)

- Pronouns  (1) 

- Gender Identity  (2) 

- Sex assigned at birth  (3) 

- Legal sex  (4) 

- Legal name  (5) 

- Preferred name  (6) 

- Something else  (7) __________________________________________________

- Decline to answer  (8) 

End of Block: Privacy

Start of Block: Patient Portal

Are patients able to update their legal sex, sexual orientation, gender identity, and or organ inventory data via patient portal?

- Yes  (1) 

- No  (2) 

- Unsure  (3) 

- Decline to answer  (4) 

Skip To: End of Block If Are patients able to update their legal sex, sexual orientation, gender identity, and or organ in... = No

Skip To: End of Block If Are patients able to update their legal sex, sexual orientation, gender identity, and or organ in... = Decline to answer

What are patients able to update via patient portal? (check all that apply)

- Gender identity  (1) 

- Sex assigned at birth  (2) 

- Sexual orientation  (3) 

- Legal sex  (4) 

- Organ Inventory  (5) 

- Preferred pronouns  (6) 

- Something else  (7) __________________________________________________

End of Block: Patient Portal

Start of Block: SSOGI Updates

What is your best approximation of the most recent update to the sexual orientation, gender identity data collection at your institution?

- 2015 or earlier  (1) 

- 2016  (2) 

- 2017  (3) 

- 2018  (4) 

- 2019  (5) 

- 2020  (6) 

- 2021  (7) 

- 2022  (8) 

- I don't know  (9) 

End of Block: SSOGI Updates

Start of Block: Additional comments or questions

If there is anything else you would like to tell us that is not captured in this survey, please let us know below.

________________________________________________________________

End of Block: Additional comments or questions
